# Toll-like Receptor 4 gene polymorphisms do not associate with normal tension glaucoma in a Korean population

**Published:** 2011-08-31

**Authors:** Wool Suh, Sungmin Kim, Chang-Seok Ki, Changwon Kee

**Affiliations:** 1 Department of Ophthalmology, Samsung Medical Center, Sungkyunkwan University School of Medicine, Seoul, Korea; 2 Department of Laboratory Medicine & Genetics, Samsung Medical Center, Sungkyunkwan University School of Medicine, Seoul, Korea

## Abstract

**Purpose:**

To evaluate the association of Toll-like receptor 4 (*TLR4*) gene polymorphisms with normal tension glaucoma (NTG) in the South Korean population.

**Methods:**

A total of 147 normal tension glaucoma patients in South Korea were recruited from April, 2007 to August, 2008. Allele, genotype, and haplotype of 8 different types of *TLR4* single nucleotide polymorphisms were analyzed: rs10759930, rs1927914, rs1927911, rs12377632, rs2149356, rs11536889, rs7037117, and rs7045953. Three hundred eighty healthy, unrelated South Korean adults were enrolled as controls.

**Results:**

Frequencies of the *TLR4* allele did not show any statistically significant difference between normal tension glaucoma patients and the control group (p>0.00625). The same results were observed in genotype frequency analysis. In addition, no statistically significant difference was observed in the frequency of haplotypes in cases of normal tension glaucoma when compared with controls.

**Conclusions:**

*TLR4* single nucleotide polymorphisms are not associated with normal tension glaucoma. Findings from this study suggest that *TLR4* polymorphisms may not play an important role in NTG pathogenesis in the South Korean population.

## Introduction

In the study of glaucoma, molecular genetics is still a challenging technology, but it aids in the determination of the etiology of the disease and points toward an understanding of its pathophysiology, ultimately allowing for its prevention and cure. In previous studies, genetic loci and responsible genes associated with various glaucomas have been identified: the trabecular meshwork glaucocorticoid response gene (*TIGR*/myocillin) in chromosome 1q23–25 is associated with juvenile-onset open angle glaucoma, the gene in chromosome 7q36 with pigment dispersion syndrome, and the cytochrome P4501B1 (*CYP1B1*) gene in chromosome 2p21 with primary congenital glaucoma [1]. Adult-onset chronic open angle glaucoma, the most common type of glaucoma, has also been reported to show strong evidence for genetic heterogeneity, and at least 11 genetic loci, along with 3 genes (myocilin, optineurin, and WD repeat domain 36 gene [*WDR36*]), have been identified [1].

A recent study reported that average intraocular pressure in an ophthalmologically normal Japanese population was 14.5 mmHg, lower than the value for Western countries, and prevalence of normal tension glaucoma was 92%, higher than that of other countries [2,3]. The Namil study, an epidemiology study in South Korea, also reported that the prevalence of primary open angle glaucoma was 3.9%, and in 80% of the population studied, the intraocular pressure was 21 mmHg or less [4]. These results implicate the influence of genetic background and/or environmental exposure in the development of normal tension glaucoma (NTG). Regarding genetic aspects, polymorphisms in the optic atrophy type 1 gene have been associated with NTG in some cases, and optineurin, which is in the one locus (GLC1E), has also been associated with a small fraction of cases of NTG [5,6]. Optineurin protein may function to protect the optic nerve from tissue necrosis factor α–mediated apoptosis, and loss of function of this protein may decrease the threshold for ganglion cell apoptosis in patients with glaucoma [7]. In addition, E50K mutations in optineurin, although rare, have contributed to a severe form of NTG, and studies of lymphocytes have demonstrated altered expression of the *p53* gene, a known regulator of apoptosis [8,9].

Recently, Shibuya et al. [10] suggested an association of multiple single nucleotide polymorphisms (SNPs) in Toll-like receptor 4 (*TLR4*) with a risk of NTG, and the ligands and/or cytokines involved in the TLR4 signaling network may be risk factors for the development of NTG. TLRs recognize both endogenous and exogenous molecules [11]. Arbour et al. [12] reported a close relationship between D299G, the non-synonymous change of the *TLR4* gene, and endotoxin hyporesponsiveness; its association with atherosclerosis, Crohn’s disease, and ulcerative colitis was also reported [12-14]. In the opththalmologic field, heat shock protein and lipopolysaccharide (LPS) were previously suggested as potential candidates for NTG antigens, and the TLR protein was known to recognize these [11]. C3H/HeJ mice with *TLR4* mutations showed glaucomatous optic nerve change in addition to defects of the LPS signaling pathway [15]. Despite this evidence, the role of *TLR4* as one of the risk factors for NTG development is still unknown. To this end, we have evaluated the association of *TLR4* polymorphisms with normal tension glaucoma and further investigated the phenotype-genotype correlation.

## Methods

### Subjects

One hundred forty-seven unrelated South Korean patients with NTG were recruited. The criteria applied for diagnosis of NTG were as follows: presence of glaucomatous optic neuropathy with compatible glaucomatous visual field defects on Humphrey 30–2 standard automated perimetry in association with an open angle on gonioscopy, and intraocular pressure (IOP) below 24 mmHg by Goldmann applanation tonometry without anti-glaucoma medication. A visual field test was considered abnormal if 2 of the following 3 criteria were met on at least 2 consecutive examinations with acceptable reliability standards (fixation loss <20%, false-positive rate <33%, and false-negative rate <33%): (1) an abnormal glaucoma hemifield test result (borderline findings were not considered abnormal), (2) at least 3 contiguous non-edge points (allowing 2 nasal step edge points) with p<0.05 on the pattern standard deviation plot and at least 1 point with p<0.01, and (3) a corrected pattern standard deviation with p<0.05. The location and pattern of the defect had to be consistent between the 2 consecutive visual field examinations, and the glaucomatous optic disc damage had to be consistent with the visual field abnormality. For inclusion in this study, patients were required to have untreated IOP readings of 24 mmHg or lower at all times, as measured by glaucoma specialists. Patients were excluded if they had a history of angle closure, ocular trauma, corneal opacity, laser iridotomy, inflammatory eye disease, non-glaucomatous optic neuropathy or other neuro-ophthalmic disease, or ocular surgery. Patients with pseudoexfoliation, pigment dispersion, consistently unreliable visual field tests, or best-corrected visual acuity of less than 20/40 were also excluded. All patients were followed long-term to ensure diagnosis of NTG.

Three hundred eighty unrelated healthy South Koreans who did not have glaucoma and family history of glaucoma were enrolled as the control group. They were all of Korean ethnicity, age- and sex-matched, and with no consanguineous marriage. A diagnosis of glaucoma was ruled out through examination of IOP and optic disc in all members of the control group. The study was conducted in compliance with the tenets of the Declaration of Helsinki for the use of human subjects in biomedical research, and institutional review board approval was obtained. Written informed consent was obtained from all study subjects.

### *TLR4* analysis

Genomic DNA was extracted from leukocytes of peripheral blood, and eight single nucleotide polymorphisms (SNPs; rs10759930, rs1927914, rs1927911, rs12377632, rs2149356, rs11536889, rs7037117, and rs7045953) of *TLR4* were investigated in all NTG cases and control groups. Genotyping of SNPs was performed using the 5′ exonuclease assay (TaqMan: Applied Biosystem, Inc. [ABI], Foster City, CA). The fluorescence signal of the probe was detected with Real-time Polymerase chain reaction (TaqMan assay for Real Time PCR [RT–PCR], 7000 Real Time PCR Systems; ABI).

### Phenotype analysis

Demographic data for all NTG patients were reviewed: age, sex, refractive errors, central corneal thickness using a contact-type ultrasound pachymeter (IOPac^®^, Heidelberg engineering, Heidelberg, Germany), underlying systemic disease, current medication, and follow-up periods. Baseline intraocular pressure by Goldmann applanation tonometry without anti-glaucoma medication, response of intraocular pressure to anti-glaucoma medication, and types and numbers of anti-glaucoma medication were also recorded. Mean deviation and pattern standard deviation in Humphrey 30–2 standard automated perimetry, presence of visual field progression, and changes in optic disc and retinal nerve fiber layer were also recorded.

### Statistical analysis

PASW software (version 17.0, SPSS, Inc., Chicago, IL) was used for statistical analyses. Call rates of the control and NTG groups were calculated. All SNPs among cases and control groups were assessed for Hardy–Weinberg equilibrium using a χ^2^ test. To examine the association, we used χ^2^ tests or Fisher exact tests (if needed) for comparison of the allele frequencies, genotype frequencies, and haplotypes. In the genotype trend analysis, we evaluated any change in frequency, depending on the change in the genotype. In the genotype dominance model, the frequencies of the dominant genotype were compared with other types of genotypes, and, in the recessive model, the frequencies of the minor genotype were compared with other extra types. To correct for multiple testing bias, 10,000 permutations for computation of p with the Haploview program were used. The program Haploview 3.32 was used for the computation of pair-wise linkage disequilibrium statistics, and *D*’ values expressed in percentages were plotted [16]. Haplotype frequencies were estimated using a previously mentioned accelerated expectation-maximization algorithm [17]. A p-value of less than 0.05 was considered statistically significant and Bonferroni’s correction was applied for multiple tests.

## Results

When eight SNPs in *TLR4* were genotyped, genotype distributions of all SNPs in the controls and the NTG cases exhibited Hardy–Weinberg equilibrium, and call rates of controls and NTG patients were all 100% (Table 1). Allele frequencies of the 5 SNPs in cases and controls are listed in Table 2. Minor allele frequencies of SNPs of NTG cases were lower than those of controls, except for rs11536889 (Table 2). Evaluation using Bonferroni’s correction found no statistically significant association with any of the SNPs between NTG cases and controls (p>0.05/8=0.00625).

**Table 1:** Hardy–Weinberg equilibrium test.

** **	**p**			**Call rate**		
**SNPs**	**Controls**	**Cases**	**Total**	**Controls**	**Cases**	**Total**
rs10759930	0.511	0.815	0.498	1	1	1
rs1927914	0.435	0.815	0.433	1	1	1
rs1927911	0.519	0.923	0.550	1	1	1
rs12377632	0.488	0.968	0.573	1	1	1
rs2149356	0.464	0.968	0.550	1	1	1
rs11536889	0.758	0.320	0.431	1	1	1
rs7037117	0.791	0.392	0.501	1	1	1
rs7045953	0.121	0.208	0.048	1	1	1

SNPs: single nucleotide polymorphisms; p: by χ^2^ test.

**Table 2:** Allele frequencies of SNPs of *TLR4* analysis.

** **	** **	**Minor allele frequency (%)**		** **	** **	** **	** **
**SNPs**	**Alleles (1/2)**	**Controls**	**Cases**	**p**	**p_c_**	**p_f_**	**OR (95% CI)**
rs10759930	T/C	41.7	40.1	0.641	0.781	0.675	0.93 (0.70–1.24)
rs1927914	A/G	41.5	40.1	0.669	0.88	0.676	0.94 (0.70–1.25)
rs1927911	G/A	41.0	39.7	0.709	0.974	0.727	0.94 (0.71–1.26)
rs12377632	C/T	41.4	39.4	0.555	0.983	0.576	0.92 (0.69–1.22)
rs2149356	G/T	41.1	39.4	0.608	0.986	0.625	0.93 (0.69–1.23)
rs11536889	G/C	23.5	26.5	0.312	0.988	0.337	1.17 (0.84–1.61)
rs7037117	A/G	25.6	24.8	0.781	0.992	0.813	0.95 (0.69–1.31)
rs7045953	A/G	9.47	7.82	0.401	0.999	0.472	0.81 (0.47–1.34)

SNPs: single nucleotide polymorphisms; p: by χ^2^ test; p_c_: corrected p-value using 10,000 permutations; p_f_: by Fisher-exact test; OR: odds ratio; CI: confidence interval.

Genotype frequencies of the 8 SNPs are shown in Table 3 and Table 4. Genotype trend analysis showed no statistically significant difference between controls and NTG patients (p>0.00625). In the analyses with the minor allele dominance model and the minor allele recessive model, none of the SNPs showed a statistically significant association between the two groups (p>0.00625). In a comparison of the frequencies in each genotype, no statistically significant association was found in any of the 8 SNPs (p>0.00625).

**Table 3:** Genotype frequencies of SNPs of TLR4 analysis I.

** **	** **	**Genotype frequency (n)**						** **	** **
** **	** **	**Cases**			**Controls**			** **	** **
**SNPs**	**Alleles (1/2)**	**1/1**	**1/2**	**2/2**	**1/1**	**1/2**	**2/2**	**p**	**p_f_**
rs10759930	T/C	52	72	23	126	191	63	0.884	0.913
rs1927914	A/G	52	72	23	126	192	62	0.889	0.889
rs1927911	G/A	53	71	23	129	190	61	0.900	0.897
rs12377632	C/T	54	70	23	127	191	62	0.771	0.777
rs2149356	G/T	54	70	23	128	191	61	0.800	0.804
rs11536889	G/C	77	62	8	221	139	20	0.471	0.464
rs7037117	A/G	85	51	11	211	143	26	0.814	0.820
rs7045953	A/G	126	19	2	314	60	6	0.692	0.744

SNPs: single nucleotide polymorphisms; p: by χ^2^ test; p_f_: by Fisher-exact test.

**Table 4:** Genotype frequencies of SNPs of TLR4 analysis II.

** **	**Major versus hetero genotype**		**Major versus minor genotype**		
**SNPs**	**p_f_**	**OR (95% CI)**	**p_f_**	**OR (95% CI)**	**p_t_**
rs10759930	0.746	0.913 (0.587–1.426)	0.771	0.885 (0.472–1.626)	0.636
rs1927914	0.667	0.908 (0.584–1.419)	0.771	0.899 (0.479–1.654)	0.664
rs1927911	0.668	0.909 (0.585–1.418)	0.884	0.917 (0.489–1.687)	0.704
rs12377632	0.519	0.862 (0.554–1.343)	0.666	0.872 (0.466–1.601)	0.550
rs2149356	0.520	0.869 (0.559–1.353)	0.771	0.894 (0.477–1.641)	0.603
rs11536889	0.223	1.279 (0.843–1.937)	0.822	1.147 (0.419–2.857)	0.304
rs7037117	0.606	0.885 (0.575–1.354)	1	1.050 (0.447–2.318)	0.784
rs7045953	0.495	0.789 (0.426–1.406)	1	0.831 (0.080–4.727)	0.420
** **	**Minor allele dominance model**		**Minor allele recessive model**		** **
**SNPs**	**p_f_**	**OR (95% CI)**	**p_f_**	**OR (95% CI)**	** **
rs10759930	0.681	0.906 (0.597–1.383)	0.895	0.933 (0.528–1.605)	** **
rs1927914	0.681	0.906 (0.597–1.383)	0.895	0.951(0.537–1.638)	** **
rs1927911	0.683	0.911 (0.601–1.388)	1	0.970 (0.547–1.672)	** **
rs12377632	0.475	0.864 (0.571–1.315)	0.895	0.951 (0.537–1.638)	** **
rs2149356	0.540	0.875 (0.578–1.331)	1	0.970 (0.547–1.672)	** **
rs11536889	0.240	1.263 (0.845–1.885)	1	1.035 (0.385–2.525)	** **
rs7037117	0.695	0.910 (0.607–1.361)	0.849	1.101 (0.477 −2.382)	** **
rs7045953	0.434	0.793 (0.441–1.379)	1	0.860 (0.083–4.880)	** **

SNPs: single nucleotide polymorphisms; p: by χ^2^ test; p_f_: by Fisher-exact test; p_t_: trend of genotype by χ^2^ test; OR: odds ratio; CI: confidence interval.

**Figure 1:**
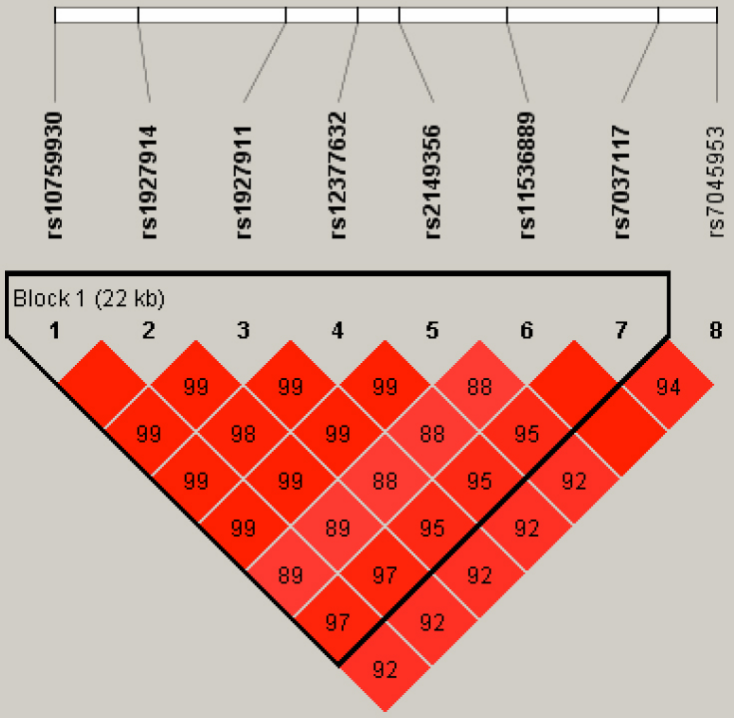
Linkage disequilibrium plot of 8 SNPs of *TLR4* in NTG patients and controls.

All 8 SNPs were located in 1 haplotype block, and the magnitude of linkage disequilibrium between each SNP was high, with pair-wise *D*≥0.85 (Figure 1). Haplotype frequencies are listed in Table 5. Frequencies of haplotypes TAGCGCA and CGATTCA were lower in NTG cases when compared with those of the controls. However, when evaluated using Bonferroni’s correction, this difference did not show statistical significance. No other significant differences in other haplotype frequencies between cases and controls were detected.

**Table 5:** Haplotype of SNPs of *TLR4* analysis.

** **	**Frequency (%)**			** **	** **
**Haplotype**	**Total**	**Cases**	**Controls**	**p**	**p_c_**
TAGCGGA	34.9	34.3	35.1	0.821	0.963
CGATTGG	24.8	23.9	25.2	0.663	0.97
TAGCGCA	23.2	24.8	22.5	0.429	1
CGATTGA	14.7	14.3	14.9	0.791	1
CGATTCA	1.2	1.5	1.0	0.472	1

SNPs: single nucleotide polymorphisms; p by χ^2^ test; p_c_: corrected p-value using 10,000 permutations.

## Discussion

Recognition of pathogens is mediated by a set of germline-encoded receptors, referred to as pattern-recognition receptors, and TLRs function as the pattern-recognition receptors in humans [18]. TLRs are related to both innate immunity and adaptive immunity through the recognition of microbial components and the induction of the production of cytokines, such as Interleukin 12 and Interleukin 18, driving the differentiation of T-cells [18]. Ten members of TLR have been reported, and *TLR* genes are dispersed throughout the genome; those encoding TLR1 and TLR4 map to human chromosome 4p14, TLR2 and TLR3 to 4q31.3-q35, TLR4 to 9q32-q33, TLR5 to 1q33.3-q42, TLR7 and TLR8 to XP 22 [18]. In particular, TLR4 recognizes the entirely unrelated ligands LPS, heat shock proteins 60 and Taxol. Among these, an association of LPS and heat shock proteins with glaucoma has been previously reported [11]. With regard to NTG, a recent study reported on an association of multiple SNPs in *TLR4* with the risk of NTG in the Japanese population [10]. The minor allele of 3 SNPs (rs10759930, rs1927914, and rs7037117) increased the risk of NTG and the minor allele of 6 SNPs (rs10759930, rs1927914, rs1927911, rs12377632, rs2149356, and rs7037117) had a 1.47 to 1.65 fold increased risk of NTG [10]. In addition, a strong association with rs7037117 was reported [10].

We attempted to determine whether there were any associations between *TLR4* polymorphisms and normal tension glaucoma in the South Korean population, and we further planned to investigate the phenotype-genotype correlation in NTG patients according to *TLR4* SNPs. However, our study did not show any association of *TLR4* SNPs with risk for NTG. There were some possible explanations for this contradictory result. One is the difference between study subjects. In our study, criteria for IOP in NTG patients was defined as 24 mmHg, whereas in the previous study it was defined as 21 mmHg with Goldmann applanation tonometry without anti-glaucoma medication. Also, the previous study group limited the range age of NTG cases as ≤20 years or ≥60 years; however, we only limited the lowest level of age to include adult-onset open angle glaucoma. The previous study focused on cases with a comparatively early onset, because early onset suggests stronger involvement of genetic factors, as the study authors mentioned. However, we did not focus on early-onset NTG, since we planned to investigate the general risk for NTG patients, regardless of age. There was also a difference in the number of study participants. The Japanese study included 255 NTG cases and 318 healthy individuals, while 147 patients with NTG and 380 healthy controls were recruited in our study group. Selection criteria for the controls in the two study groups were also different, in that control individuals in our study were age- and sex-matched, whereas controls in the Japanese group were only age-matched.

In addition to the differences in study design between the two studies, interestingly, minor allele frequencies in control subjects in our study differed from those in the Japanese study, though statistical analysis was not performed (Table 2). Except for rs7037117, frequencies of minor alleles of controls in the Japanese group were lower than those of our study results. Furthermore, there was also a difference in genotype frequencies in controls between the two populations (Table 3, Table 4, and Table 5). These differences in the control group may have influenced the study results. Adult-onset forms of glaucoma, particularly normal tension glaucoma, are inherited as complex traits, which is likely not inherited as a single gene. Thus, different environmental factors, including ethnicity in two populations or their genetic backgrounds, involving the interference of multiple genes, may have influenced the results.

In the beginning of the study, we planned to evaluate the genotype-phenotype correlation in NTG patients. Following Wiggs, we had three research questions in mind: (1) What is the range of phenotypic variation of a given mutation, i.e., can one predict the prognosis of the disease knowing the specific mutation responsible for the disease?; (2) Are certain mutations associated with particular aspects of the disease phenotype?; and (3) Are certain mutations necessary but not sufficient to cause the disease [19]. However, because there were no statistically significant associations between *TLR4* SNPs and NTG, we did not investigate further the aspects of the relationship between NTG phenotype and *TLR4* SNPs.

In conclusion, *TLR4* single nucleotide polymorphisms are not associated with normal tension glaucoma. This result suggests that *TLR4* polymorphisms may not play an important role in NTG pathogenesis in the South Korean population. A few studies have been conducted for identification of the genetic risk, especially *TLR4* SNPs, as a possible risk factor of NTG. Although we considered genetic and environmental variables, results differ between different groups of patients. Thus, further studies need to be replicated repeatedly to confirm the association of *TLR4* SNPs and normal tension glaucoma. Moreover, we still do not know how TLRs recognize entirely unrelated ligands, and how this functions in human cells. Thus, a more basic molecular biologic approach to TLRs will be needed in the future.
